# Hidden Order in the Apparent Chaos of Bias Temperature Instability

**DOI:** 10.3390/mi17080903

**Published:** 2026-07-28

**Authors:** Joseph B. Bernstein

**Affiliations:** Department of Electrical and Electronic Engineering, Ariel University, Ariel 40700, Israel; josephbe@ariel.ac.il

**Keywords:** reliability physics, degradation kinetics, Gibbs free energy, degradation correlation, power–law degradation, bias temperature instability (BTI), electromigration, lifetime prediction

## Abstract

Bias Temperature Instability (BTI) remains one of the principal reliability challenges limiting advanced CMOS technologies. Although degradation is commonly described by an empirical power–law relationship, the power–law exponent is generally regarded only as a fitting parameter used for lifetime extrapolation. This Perspective reexamines a previously published Multiple-Temperature Operational Life (MTOL) dataset to investigate whether the measured exponent contains previously overlooked physical information. Individual ring oscillators stressed under identical voltage and temperature conditions exhibit substantially different, yet reproducible, power–law exponents. When these measurements are analyzed over a broader temperature range, the apparent statistical scatter reveals a systematic kinetic dependence that produces a remarkably consistent lifetime relationship after incorporating the experimentally measured exponent into the Arrhenius analysis. The resulting intrinsic activation energy is significantly smaller than values obtained using conventional extrapolation methods, suggesting that part of the apparent activation energy arises from neglecting the temperature dependence of the degradation exponent. A recently proposed thermodynamic formulation based on Gibbs free energy and correlation entropy is presented as one possible physical interpretation of these observations, in which the power–law exponent reflects the correlation between successive degradation events rather than merely an empirical fitting constant. More generally, this Perspective suggests that the power–law exponent should be regarded as a measurable kinetic quantity whose systematic variation may provide additional insight into degradation mechanisms in BTI and other reliability phenomena.

## 1. Introduction

Over the past few decades, Bias Temperature Instability (BTI) has become one of the principal degradation mechanisms limiting advanced CMOS technologies [1,2]. During the same period, transistor density and computational performance have continued to increase at an extraordinary pace while operating voltage, timing, and thermal design margins have continued to shrink. Modern integrated circuits therefore operate closer to their physical limits than ever before.

The recent growth of artificial intelligence and hyperscale computing have made this problem even more important. Today’s processors contain billions, or in some cases trillions, of transistors operating continuously under demanding electrical and thermal conditions. Small changes in transistor degradation can therefore influence performance, power consumption, and ultimately system reliability. As operating margins continue to shrink, accurate prediction of degradation is no longer simply a lifetime problem. It has become an important design problem.

The importance of BTI is reflected by the remarkable growth of research devoted to understanding its physical origin and predicting device lifetime. As illustrated by Stathis [3], the number of BTI publications has increased dramatically over the past 20 years. During this period, experimental techniques, compact models, atomistic simulations, and physical interpretations have all advanced significantly [4,5,6,7]. Nevertheless, several important questions remain. The physical origin of BTI is still debated. Reported activation energies span a surprisingly wide range, and even the interpretation of the empirical power–law exponent remains uncertain [3,8,9].

Most BTI studies describe degradation using the empirical power–law relationship [1,10,11,12]

(1) $$\Delta P=A(V,T)t^{n}$$

where ΔP represents the measured degradation parameter, such as threshold-voltage shift, $\Delta V_{t}$, or ring oscillator frequency shift, Δf. The exponent n is obtained by fitting the experimental data. This empirical relationship has proven remarkably successful across many technologies and stress conditions. However, the exponent itself is usually regarded only as a fitting parameter. Whether the exponent carries measurable physical information has received surprisingly little attention.

One of the earliest comprehensive assessments of the problem was presented by Alam [2], who observed that “…none of the existing NBTI models are comprehensive enough to consistently explain…” the growing body of experimental observations. This challenge motivated the development of increasingly sophisticated physical models together with improved experimental techniques. Schroder later emphasized the need for a more consistent physical interpretation of the measured degradation kinetics [8], while Grasser and co-workers described a paradigm shift from classical reaction–diffusion models toward switching oxide traps [5].

More recently, fast measurement techniques, improved methodologies, and advanced FinFET and gate-all-around technologies have substantially improved both the quality of BTI measurements and the understanding of degradation physics [4,6,7]. Nevertheless, the measured activation energy and power–law exponent continues to exhibit considerable variation among different technologies and experimental conditions. As noted by Stathis, Mahapatra, and Grasser, “Merely demonstrating the ability to fit data does not constitute such proof” [13]. This observation is particularly relevant to empirical power–law degradation models, where the exponent is routinely extracted but its physical significance is rarely examined. Previous studies have likewise reported that power–law degradation is consistently observed over a wide range of BTI stress conditions, although with different values of the time exponent, suggesting that the exponent itself contains reproducible physical information rather than merely fitting uncertainty [4].

Recent investigations in advanced FinFET and gate-all-around technologies have further demonstrated that the measured power–law exponent varies systematically with technology, stress conditions, and extraction methodology [6,7]. At the same time, growing attention has been devoted to the interpretation of activation energies in BTI and to the limitations of extracting physical mechanisms solely from empirical fitting parameters [9,13]. The present Perspective extends these observations by proposing that the experimentally measured power–law exponent should itself be regarded as a measurable kinetic quantity rather than merely an empirical fitting parameter used for lifetime extrapolation.

**Figure 1:**
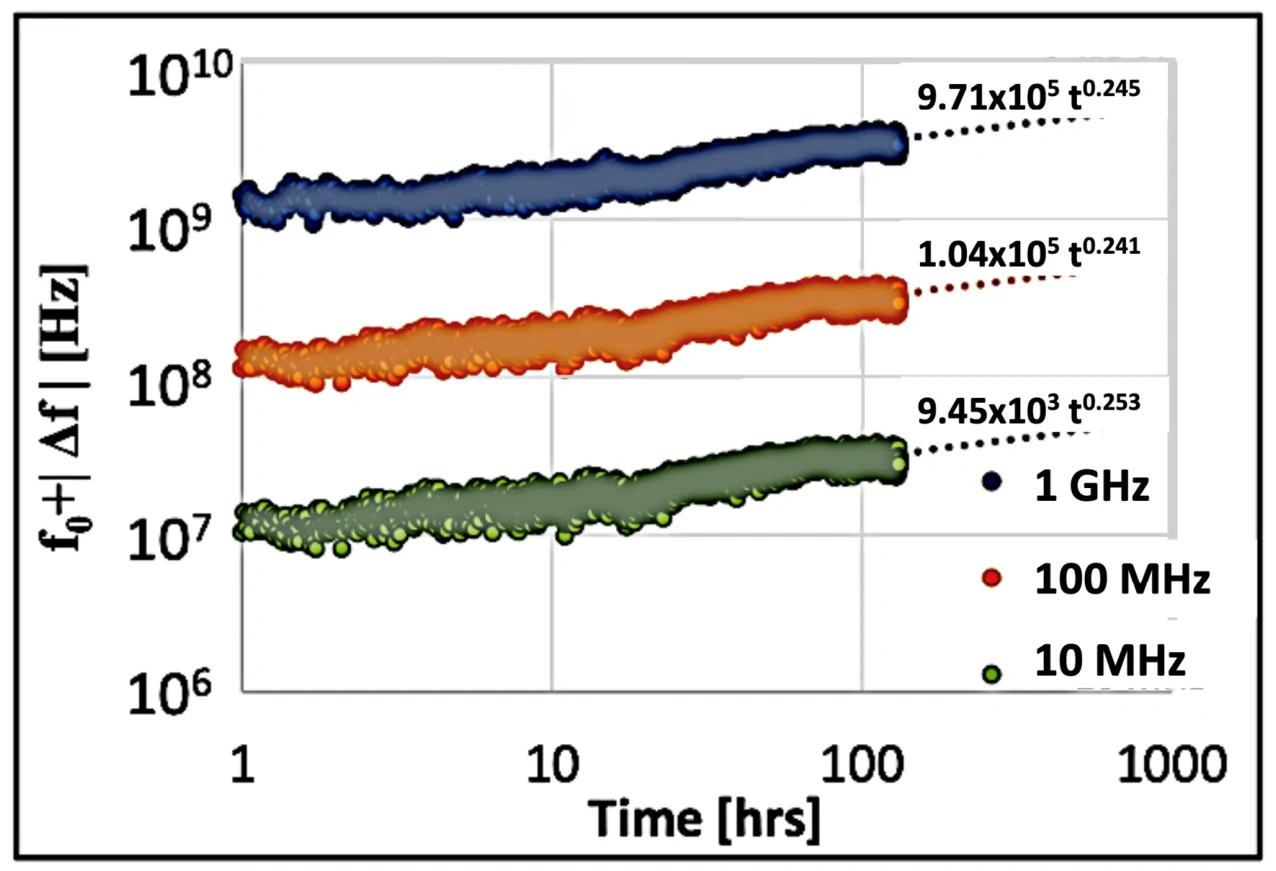
Traditional log–log representation of frequency degradation across ring oscillators of different operating frequencies. The subtle upward curvature can lead to substantial lifetime extrapolation errors when a single population exponent is used for lifetime extrapolation.

The present work revisits a previously published dataset related to BTI degradation in a 16 nm FinFET technology [11]. Figure 1 illustrates the standard industry approach, plotting frequency degradation (Δf) over time across multiple operating frequencies. Traditional analysis relies on a critical assumption: that a collective population average accurately models individual circuit wear-out. The subtle upward curvature at long stress times in Figure 1 is routinely dismissed as measurement noise or misattributed to accelerating wear mechanisms. However, a single, forced population exponent can introduce substantial error for long-term lifetime extrapolation.

## 2. Hidden Kinetic Information

Standard qualification methodologies assume that a single power–law exponent can adequately represent population-level BTI. This conventional averaging technique provides convenient empirical fits for qualifications, but it can obscure the deterministic wear-out kinetics of individual devices. A true physical model of BTI requires tracking the specific exponent of individual components rather than relying on a collective macro-estimate.

**Figure 2:**
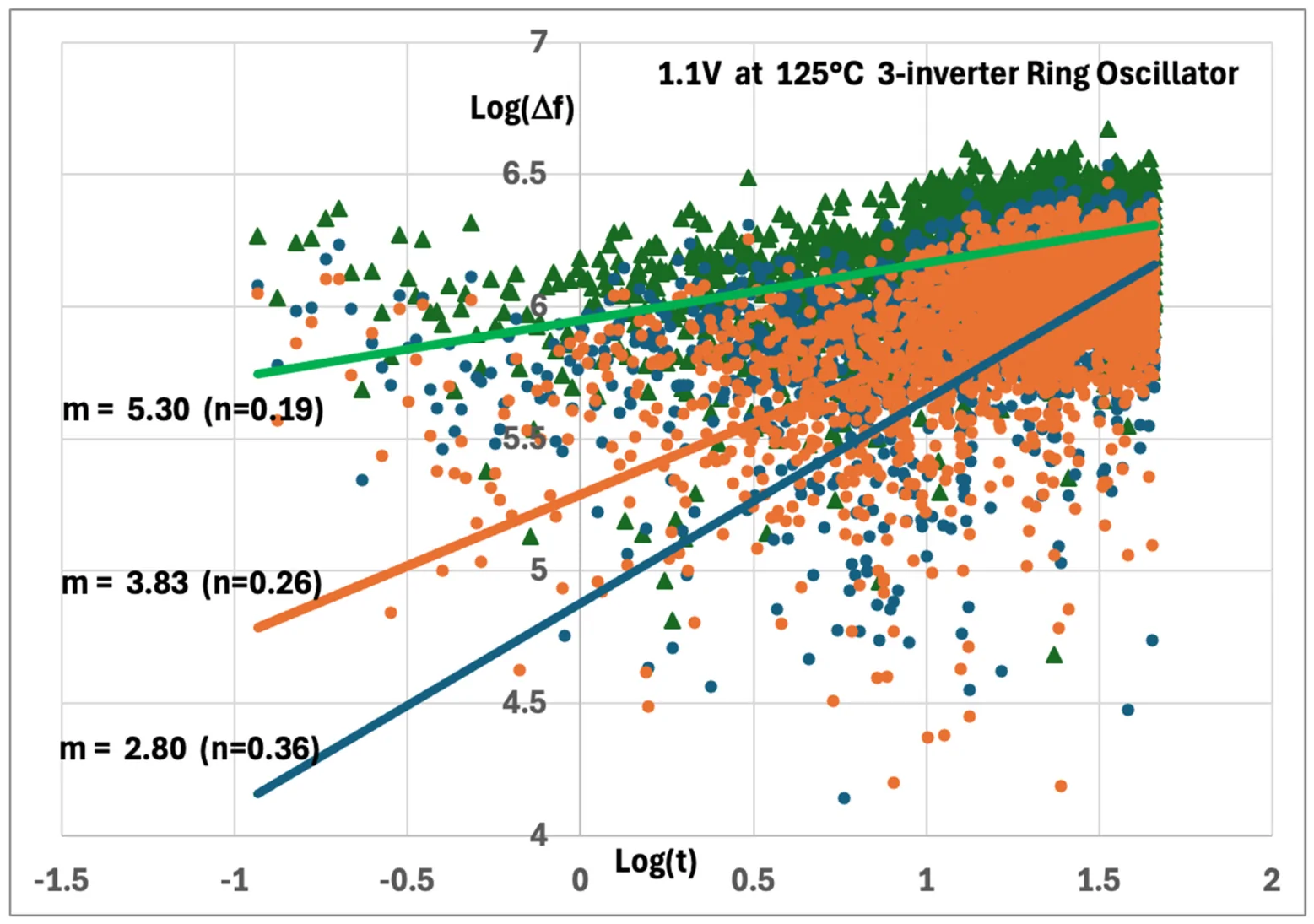
Linearized degradation trajectories for three nominally identical ring oscillators located on the exact same die and subjected to identical stress conditions. A clear resolution of baseline ambiguity reveals distinct, independent, power–law slopes for nearly identical circuits.

**Figure 3:**
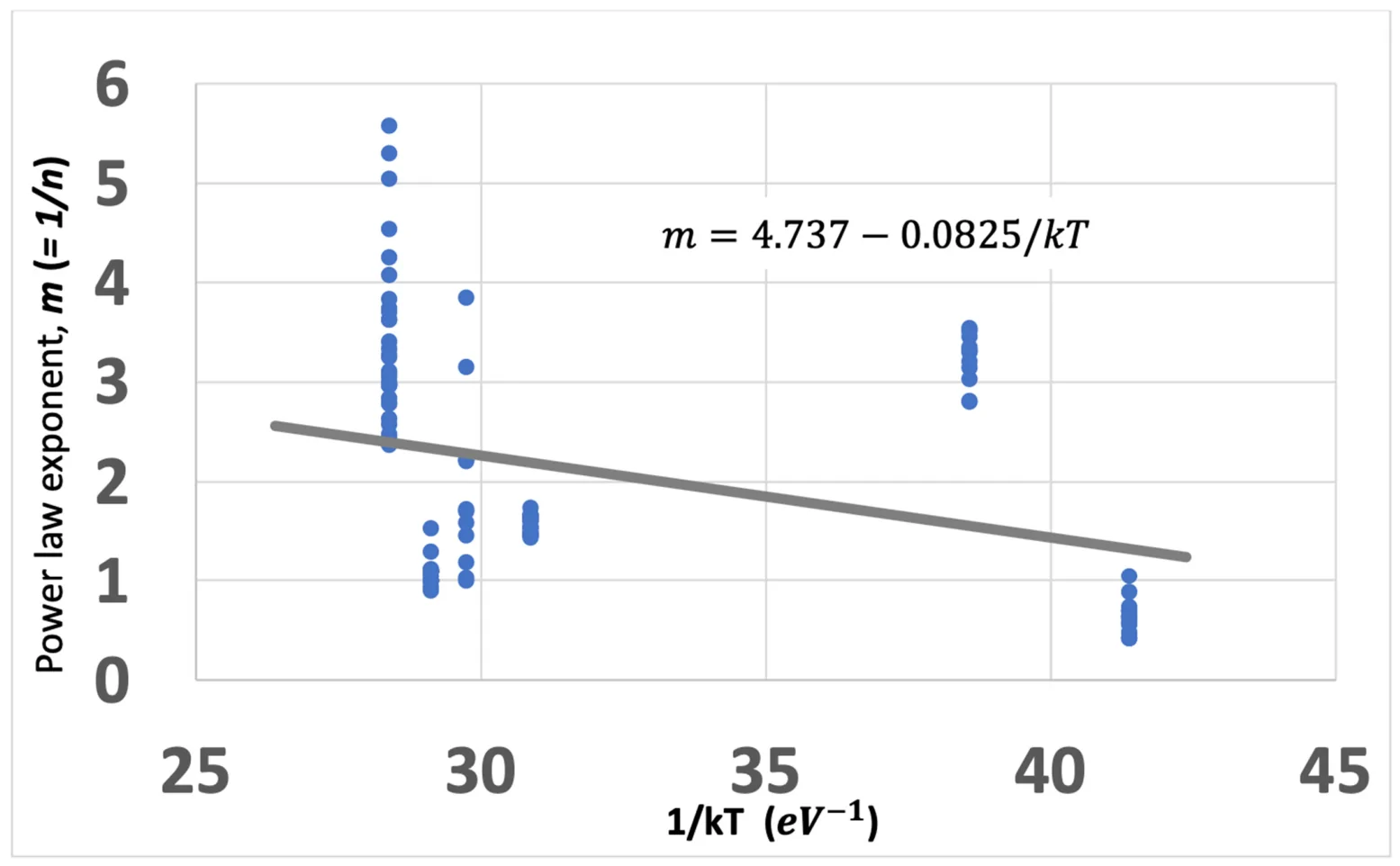
Extracted power–law exponents for individual ring oscillators as a function of reciprocal temperature. The individual measurements exhibit substantial ring-to-ring variability, while the linear regression of the average behavior reveals a systematic temperature dependence of the power–law exponent.

A mathematical correction for baseline ambiguity challenges the assumption of a universal population exponent [12]. When these individual trajectories are linearized, the apparent population consensus is no longer sufficient. Figure 2 demonstrates this phenomenon using three adjacent, nominally identical ring oscillators located on the exact same die and subjected to identical stress conditions. The three ring oscillators shown in Figure 2 were selected as representative examples illustrating the range of observed degradation trajectories. Similar device-to-device differences were observed throughout the complete dataset summarized in Figure 3.

At first inspection, the raw data forms dense clouds of statistical scatter with a remarkably similar “beehive” appearance, offering little visual confidence that meaningful differences can be extracted. This linearization procedure minimizes the residual second-order curvature introduced by the logarithmic transformation without altering the raw experimental scatter. This mathematical resolution reveals an important physical observation: despite experiencing identical global stress, each circuit degrades according to its own unique, power–law slope.

As shown by the individual trajectories in Figure 2, the extracted degradation parameters differ substantially, yielding values of m = 5.30, 3.83, and 2.80. For each fit, the residual quadratic coefficient was reduced to within ±0.005 for each. These correspond to true power–law exponents of n=0.19, 0.26, and 0.36, respectively. Individual devices subjected to identical stress conditions exhibit distinct degradation trajectories characterized by fundamentally different power–law exponents. Any qualification methodology that groups these independent responses into a singular population average may obscure the underlying physics of failure.

The power–law exponents were extracted using the previously published linearization procedure [12], in which the quadratic ($x^{2}$) coefficient of the transformed data is iteratively minimized. For all devices analyzed, the residual quadratic coefficient after optimization was reduced to within approximately ±0.005, with the exponent determined to three decimal places. As discussed previously [12], a broad range of nearby exponents produces similarly high values of $R^{2}$, making the coefficient of determination an insensitive criterion for identifying the optimum exponent. The residual-curvature minimization provides substantially greater numerical sensitivity. Consequently, the observed differences between the extracted exponents are significantly larger than the numerical resolution of the extraction procedure and represent reproducible differences in the extracted power–law exponents rather than fitting uncertainty.

At this stage no physical interpretation is implied. The sole conclusion is that individual devices subjected to identical stress conditions exhibit reproducible degradation trajectories characterized by distinctly different power–law exponents. The following section examines whether these apparently independent measurements exhibit systematic behavior when analyzed over a wider range of temperatures and operating conditions.

## 3. Emergence of Hidden Order

The previous section demonstrated that individual ring oscillators stressed under identical voltage and temperature conditions exhibit reproducible, yet significantly different, power–law exponents. At first inspection these differences appear to represent ordinary statistical variation. The next question is whether these apparently independent measurements exhibit systematic behavior over a broader range of operating conditions.

Figure 3 summarizes the extracted values of the power–law exponent for all measured ring oscillators as a function of reciprocal temperature. The considerable vertical scatter is not the result of poor fitting or measurement uncertainty alone but reflects the same ring-to-ring variability illustrated in Figure 2. Each point therefore represents a reproducible degradation trajectory for an individual device obtained under a particular voltage and temperature stress condition.

Despite this substantial variability, a systematic temperature dependence becomes apparent. Although the individual measurements do not fall directly on a single line, the average value of the extracted exponent exhibits a clear trend with reciprocal temperature. Thus, the variability observed in Figure 2 should not be interpreted as obscuring the underlying physics. Rather, it represents a statistical distribution superimposed upon an ordered kinetic behavior.

It is important to distinguish between measurement uncertainty and physical variability. Each data point in Figure 3 represents the independently extracted power–law exponent of a single ring oscillator, determined using the procedure described in the previous section. The vertical spread therefore reflects the natural distribution of reproducible device-to-device degradation trajectories rather than repeated measurements of the same device. Consequently, the objective is not to estimate the uncertainty of an average exponent, but to determine whether an ordered dependence emerges from the ensemble of independently measured exponents.

This observation is important for two reasons. First, it demonstrates that the extracted power–law exponent is not simply an arbitrary fitting parameter but a reproducible kinetic quantity. Second, it suggests that much of the apparent statistical variability observed in BTI degradation is accompanied by an underlying temperature dependence that becomes evident only after examining the complete population of measurements.

Figure 3 provides the first indication that the apparent statistical variability identified in Figure 2 is not entirely random. Although the extracted values of the power–law exponent exhibit considerable ring-to-ring variation, the overall temperature dependence is remarkably consistent. A linear regression of the average behavior yields an activation energy of approximately 0.08 eV, which is substantially smaller than values commonly reported using conventional lifetime extrapolation methods.

At first glance, the scatter in Figure 3 might appear to weaken this conclusion. In fact, the opposite is true. The vertical spread is simply the cumulative manifestation of the individual ring-to-ring variations demonstrated previously in Figure 2. The important observation is not that every data point lies on the fitted line, but that a clear temperature dependence remains evident despite this variability. The underlying kinetic trend therefore survives the statistical spread.

The significance of this result becomes apparent when compared with conventional Arrhenius analysis. Most BTI studies determine the activation energy directly from the measured time-to-failure, implicitly if the power–law exponent remains constant. In the present dataset, however, the exponent itself varies systematically with temperature. Ignoring this variation therefore attributes part of the measured temperature dependence to the apparent activation energy, producing a substantially larger value than obtained when the measured exponent is explicitly included in the analysis.

The original MTOL study assumed a representative value of the power–law exponent, resulting in an apparent activation energy of approximately 0.49 eV. When the experimentally measured exponents are incorporated into the analysis, the effective activation energy decreases to approximately 0.08 eV while simultaneously reducing the overall scatter of the lifetime extrapolation. Although the exact numerical value is naturally dataset dependent, the comparison demonstrates that accounting for the measured degradation kinetics can significantly influence the extracted activation energy.

To facilitate a unified description of the degradation kinetics, the experimentally measured exponent is expressed in terms of the correlation coefficient introduced in the recent thermodynamic formulation [14],

(2) χ=1−m

where positive and negative values of χ correspond to cooperative and self-limiting degradation kinetics, respectively, while χ=0 reduces to the conventional Arrhenius description. Since χ is dimensionless, the product χkT has units of energy and provides a convenient normalized kinetic variable for comparing different degradation mechanisms [14]. The physical interpretation of this correlation energy is discussed in the following section.

**Figure 4:**
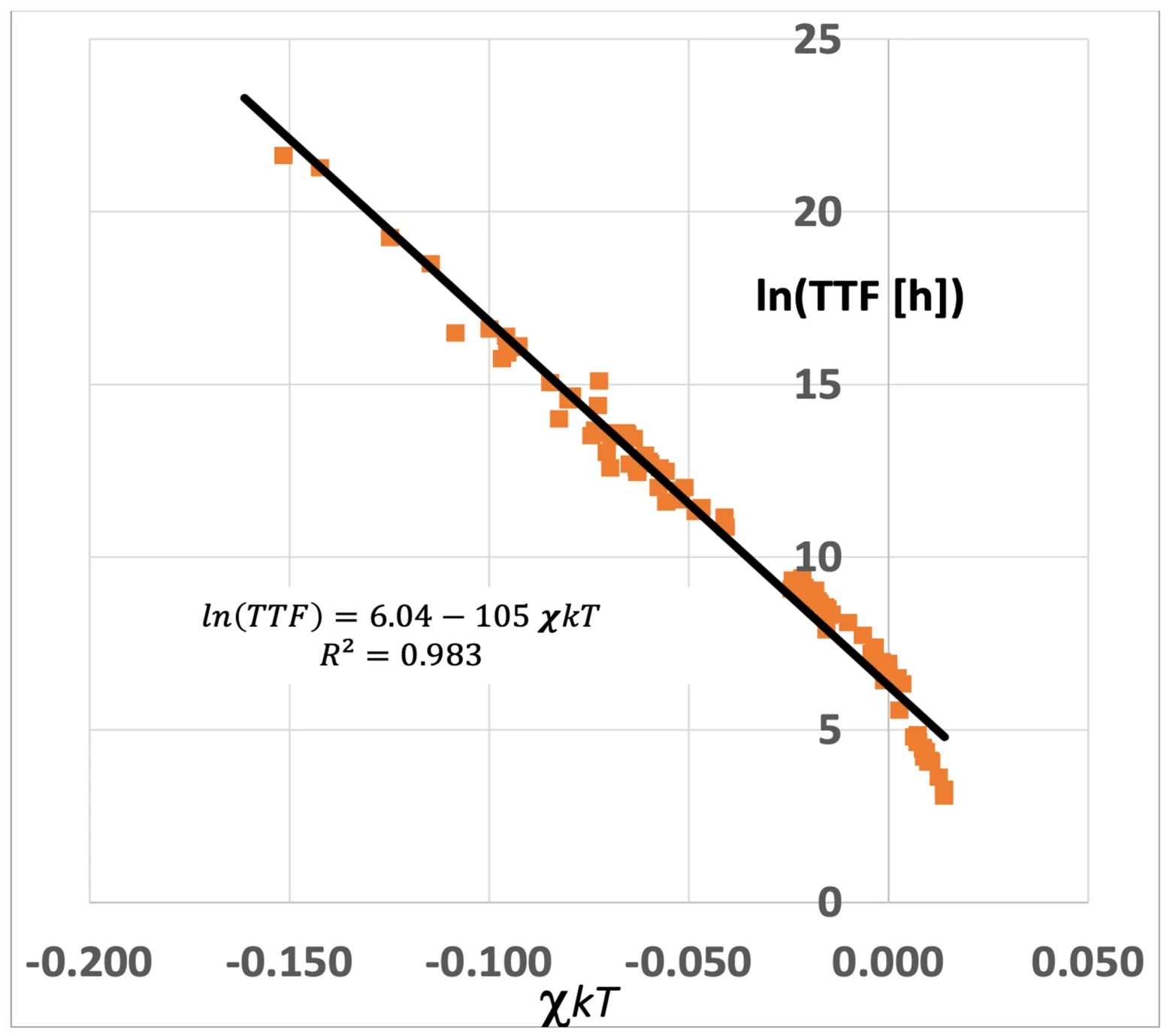
Unified Arrhenius lifetime collapse obtained by incorporating the experimentally measured power–law exponent into the temperature acceleration analysis.

Figure 4 is a replotting of the lifetime data using the normalized variable χkT. The improvement is immediately apparent. Data that previously exhibited substantial scatter now collapse onto a consistent kinetic relationship while preserving the experimentally measured variation in the power–law exponent. Figure 4 therefore demonstrates the consequence of incorporating the experimentally measured power–law exponent into the conventional Arrhenius analysis. The apparent disorder observed throughout the previous figures is therefore not eliminated but rather organized into a unified description of the degradation process.

This result is consistent with the interpretation of the extracted power–law exponent as a measurable kinetic quantity rather than simply an empirical fitting parameter. The ring-to-ring variability observed in Figure 2, together with the systematic temperature dependence demonstrated in Figure 3, produces a substantially more consistent lifetime extrapolation when the experimentally measured exponent is included in the analysis. The collapse represents more than a reduction in statistical scatter because it is obtained by incorporating the experimentally measured degradation exponent into the lifetime projection. These observations suggest that the apparent statistical variability contains reproducible physical information.

Because both the lifetime projection and the normalized variable χkT depend on the experimentally extracted exponent m, the correlation shown in Figure 4 should not be interpreted as an independent validation. Rather, it demonstrates that incorporating the measured exponent produces a self-consistent thermodynamic description of the MTOL dataset.

Perhaps the most significant observation is that the resulting activation energy is approximately 0.08 eV, substantially smaller than values commonly obtained using conventional lifetime extrapolation methods. This value is remarkably close to the low-energy activation processes reported in recent BTI investigations [6,9,15], where the apparent activation energy has been shown to depend strongly on the measurement methodology, stress conditions, and physical interpretation. The present analysis suggests that part of this variation may arise because conventional Arrhenius extrapolation implicitly assumes a constant power–law exponent. When the experimentally measured exponent is incorporated directly into the lifetime analysis, the intrinsic temperature dependence is substantially reduced.

The progression from Figure 2, Figure 3 and Figure 4 therefore reveals a consistent picture. What initially appears to be little more than statistical scatter evolves into a reproducible temperature dependence and ultimately into a unified kinetic description. The apparent chaos has not disappeared. Rather, it has been shown to contain hidden order.

## 4. Discussion

The experimental observations presented in the previous section demonstrate that the apparent statistical variability observed in BTI degradation contains considerably more information than is immediately evident from conventional power–law fitting. The progression from Figure 2, Figure 3 and Figure 4 shows that ring-to-ring variability, although substantial, is accompanied by a systematic temperature dependence that ultimately produces a unified lifetime extrapolation when the experimentally measured power–law exponent is incorporated into the analysis. The immediate question is therefore not whether the power–law exponent varies, but rather what physical significance should be attributed to that variation.

### 4.1. The Physical Meaning of the Power–Law Exponent

Historically, the power–law exponent, n=1/m, has been regarded primarily as an empirical fitting parameter. Once the degradation data are adequately represented by Equation (1), the exponent is generally used only to extrapolate lifetime and is seldom discussed further. Most physical interpretation instead focuses on the activation energy, the voltage acceleration factor, or the underlying microscopic degradation mechanism. Consequently, the possibility that the exponent itself may contain measurable kinetic information has received relatively little attention.

The present results suggest a different perspective. The extracted values of the power–law exponent are reproducible for individual devices, exhibit a systematic dependence on temperature, and significantly improve the consistency of the Arrhenius lifetime extrapolation when incorporated directly into the analysis. These observations suggest that the exponent behaves as a measurable kinetic parameter rather than merely an adjustable fitting constant.

This conclusion is also consistent with the broader BTI literature. Numerous investigators have reported significant variations in the measured power–law exponent among different technologies, stress conditions, and experimental methodologies [9,16,17]. These variations have generally been regarded as secondary observations associated with differences in degradation mechanisms or measurement techniques. The present work suggests that they may instead represent an intrinsic component of the degradation kinetics.

It is important to emphasize that the present interpretation does not depend on any specific microscopic model of BTI. Whether degradation is attributed primarily to reaction–diffusion, charge trapping, switching oxide traps, or a combination of interacting mechanisms is not the central issue. The experimental observation remains that the measured power–law exponent carries reproducible information that influences the extracted degradation kinetics. Any comprehensive physical description should therefore account not only for the measured degradation itself, but also for the systematic behavior of the exponent describing its evolution.

### 4.2. Implications for Activation Energy

The improved lifetime extrapolation shown in Figure 4 follows directly from the degradation kinetics described by Equation (1). Rather than treating the power–law exponent as an empirical fitting parameter, the present analysis begins with the measured degradation relationship for a specified failure criterion, $\Delta P=\Delta P_{crit}$, the corresponding time-to-failure becomes

(3) $$TTF={\left(\frac{\Delta P_{crit}}{A(V,T)}\right)}^{1/n}.$$

Taking the natural logarithm gives

(4) $$n\;ln(TTF)=ln(\Delta P_{crit})-ln[A(V,T)].$$

The temperature dependence is contained within the degradation coefficient $A\left(V,T\right)$, which follows the conventional Arrhenius relationship

(5) $$A\left(V,T\right)=A_{0}\;exp\left(-\frac{E_{a}}{kT}\right)\;\;.$$

Substituting Equation (5) in Equation (4) yields

(6) $$n\;ln(TTF)=\frac{E_{a}}{kT}+C\;\;\;,$$

where $C=ln(\Delta P_{crit})-ln(A_{0})$ is independent of temperature.

Equation (6) forms the basis of the Multiple Temperature Operational Life (MTOL) formulation developed previously for BTI lifetime extrapolation assuming a non-constant power–law exponent [18]. Unlike the conventional Arrhenius analysis, the experimentally measured degradation exponent is incorporated directly into the lifetime extrapolation. Since the exponent is obtained independently from the degradation kinetics, no additional fitting parameters are introduced into the analysis.

The significance of Equation (6) is illustrated by Figure 4. Conventional Arrhenius analysis assumes that the exponent remains constant, implicitly attributing all the measured temperature dependence to the activation energy. The present results demonstrate that this assumption is not generally valid. Because the degradation exponent varies systematically with temperature, part of the apparent temperature dependence is contained within the degradation kinetics themselves. When the experimentally measured exponent is incorporated into the Arrhenius formulation, the kinetic contribution associated with its temperature dependence is removed. The data therefore collapse onto a substantially more consistent Arrhenius relationship.

For the present dataset, the apparent activation energy decreases from approximately 0.49 eV using the conventional analysis to approximately 0.08 eV when the experimentally measured exponent is incorporated into the extrapolation. No additional fitting parameters are introduced. The reduction follows directly from the experimentally measured degradation kinetics. Figure 4 therefore represents more than an improved mathematical transformation. It demonstrates that the systematic variation in the degradation exponent contributes directly to the apparent activation energy extracted from accelerated lifetime measurements.

This observation may also help explain the remarkably broad range of activation energies reported throughout the BTI literature. Published values span approximately 0.1 eV to more than 1 eV and have generally been attributed to differences in technology, processing, stress conditions, or competing degradation mechanisms. While these factors undoubtedly contribute, the present work suggests an additional source of variability. If the degradation exponent itself varies systematically with temperature, analyses that assume a constant exponent will naturally extract different effective activation energies.

The improved lifetime extrapolation demonstrated in Figure 4 therefore provides evidence that the activation energy and the power–law exponent are not independent empirical fitting parameters. Instead, both quantities reflect the underlying degradation kinetics. The remaining question is therefore not whether the degradation exponent contains physical information, but what physical process gives rise to its systematic behavior. One possible interpretation is discussed in the following section.

### 4.3. Possible Physical Interpretation

One possible interpretation has recently been proposed using a thermodynamic formulation based on Gibbs free energy and correlation entropy [14]. In this framework, the apparent power–law exponent is interpreted as a measure of the correlation between successive degradation events rather than simply as an empirical fitting parameter. The degradation free energy is written as

(7) $$\Delta G=E_{a}-kT(1-m)lnN={\;E}_{a}+kT\chi lnN,$$

where N represents the accumulated degradation state and χ=1−m is the correlation coefficient introduced in Equation (2). When χ=0, the conventional Arrhenius equation is recovered. Negative values of χ correspond to self-limiting degradation, while positive values describe cooperative or accelerating degradation.

Equation (7) provides a possible physical interpretation of the experimental observations presented in this work. Rather than viewing the power–law exponent as an empirical fitting parameter, the thermodynamic formulation interprets it as the consequence of an evolving free-energy landscape during degradation. As damage accumulates, the effective degradation barrier changes continuously, naturally producing power–law kinetics while reducing to the conventional Arrhenius relationship when successive degradation events become statistically independent $\left(\chi =0\right)$.

This interpretation is also consistent with several important experimental observations established throughout the BTI literature. First, the intrinsic activation energy extracted after incorporating the experimentally measured power–law exponent is only approximately 0.08 eV, indicating remarkably weak underlying temperature dependence. Second, the power–law behavior is established from the earliest measurable stress times rather than emerging only after prolonged stressing. Third, individual devices exhibit a broad but reproducible distribution of measured power–law exponents despite nominally identical stress conditions. Together, these observations suggest that the exponent represents an intrinsic property of the degradation kinetics rather than simply statistical fitting uncertainty.

The atomistic dimensions of modern CMOS technologies provide an additional physical constraint. Typical interface-state densities are on the order of $10^{10}$ to $10^{12}\;cm^{-2}$, while the density of Si-H bonds at the ${Si/SiO}_{2}$ interface is approximately $10^{14}\;cm^{-2}$ [19]. Modern FinFET and gate-all-around transistors therefore contain only a finite population of interface defects and passivated Si-H bonds. Degradation consequently evolves through the statistical behavior of a limited number of atomic-scale defects rather than within a continuous medium. This observation becomes increasingly important as transistor dimensions approach only a few tens of nanometers, where the total number of participating defects may itself become statistically significant. Any microscopic description of BTI should therefore remain consistent with this finite defect population while simultaneously accounting for the immediate establishment of the observed power–law kinetics [16].

These observations do not invalidate existing physical models, including reaction–diffusion, charge trapping, or switching oxide trap descriptions. Rather, they establish experimental criteria that any comprehensive physical theory should satisfy. As emphasized by Stathis, Mahapatra, and Grasser, agreement with experimental fitting alone does not constitute proof of a physical mechanism [13]. A successful physical theory should therefore satisfy the experimental observations identified above while simultaneously providing a mechanistic explanation for them. The model should account for the systematic behavior of the power–law exponent, the improved lifetime extrapolation obtained by incorporating the measured exponent, the remarkably small intrinsic activation energy, and the immediate establishment of the observed power–law kinetics.

Whether the present thermodynamic interpretation ultimately proves to be the correct microscopic description remains to be established. Nevertheless, it provides a unified framework that naturally connects the principal experimental observations presented here while remaining fully consistent with the published degradation kinetics. More importantly, the present work demonstrates that what initially appears to be statistical chaos contains reproducible physical information. The apparent disorder reflects hidden kinetic order that any successful microscopic description of BTI should ultimately explain.

### 4.4. Broader Implications

The principal implication of the present work extends beyond BTI itself. The analysis presented here suggests that the power–law exponent should not be regarded simply as a convenient fitting parameter, but as a measurable kinetic quantity that may provide insight into the underlying degradation mechanism. Consequently, future reliability studies should consider the degradation exponent as an experimentally observable parameter in its own right, together with the activation energy and the voltage acceleration factor.

This perspective is applicable to many degradation mechanisms that exhibit empirical power–law behavior. In hot-carrier injection (HCI), systematic variations in the power–law exponent have long been reported under different voltage and temperature stress conditions [15,20,21]. Rather than treating these variations solely as empirical fitting differences, they may contain information regarding the degree of interaction between successive degradation events. Similarly, time-dependent dielectric breakdown (TDDB) frequently exhibits nearly independent defect generation, suggesting behavior approaching the limiting case of χ ≈ 0 [22]. Electromigration, in contrast, is characterized by progressive damage accumulation and may therefore be expected to exhibit positive correlation coefficients associated with cooperative degradation [23].

The present thermodynamic description does not replace the existing microscopic models developed for these mechanisms. Reaction–diffusion models, charge trapping, switching oxide traps, percolation theory, atomistic diffusion, and fracture mechanics remain essential for identifying the underlying physical processes [1,2,3,4,5]. Instead, the experimentally measured power–law exponent provides an additional kinetic observable against which these models may be evaluated. A successful physical description should not only reproduce the measured degradation but also explain the experimentally observed values and systematic variation in the power–law exponent [4,13].

This interpretation also suggests new experimental approaches. Rather than reporting only average degradation curves and extracted activation energies, future studies should examine the statistical distribution of power–law exponents, their dependence on voltage and temperature, and their correlation with material properties and device architecture. Such measurements may provide additional insight into degradation mechanisms that is not evident from conventional lifetime extrapolation alone.

A broader discussion of the thermodynamic interpretation of degradation kinetics and its application to multiple semiconductor reliability mechanisms has recently been presented in a tutorial review [24,25]. The objective of the present work is considerably more focused. Using a previously published BTI dataset, it demonstrates that apparently chaotic statistical scatter contains reproducible kinetic information that becomes evident when the degradation exponent is treated as a measurable physical quantity. This observation provides experimental support for applying the broader thermodynamic interpretation to reliability studies while suggesting practical methods for extracting additional physical information from conventional degradation measurements.

The data analyzed in the present study are from a previously published MTOL dataset and therefore inherit the limitations of that experimental methodology. The extracted power–law exponent may be influenced by process variability, baseline selection, fitting-window dependence, recovery effects, and the simultaneous contribution of multiple degradation mechanisms. Although these factors may affect the numerical value of the extracted exponent, the present results demonstrate that systematic kinetic information can nevertheless emerge from the ensemble of experimentally measured degradation trajectories. Future studies using additional technologies and controlled stress conditions will be valuable for assessing the generality of the proposed framework.

## 5. Conclusions

The present work revisited a previously published BTI dataset with a different objective from the original MTOL investigation. Rather than focusing solely on accurate lifetime extrapolation, the analysis examined whether the apparent statistical variation in the measured power–law exponent contains previously overlooked physical information. The results demonstrate that the apparent disorder observed in individual degradation measurements conceals remarkably consistent kinetic behavior.

Individual ring oscillators stressed under identical electrical and thermal conditions exhibit reproducible power–law exponents that differ significantly from one another. Although this variability initially appears to represent ordinary statistical scatter, a systematic temperature dependence emerges when the complete dataset is examined. The power–law exponent therefore behaves as a measurable kinetic quantity rather than simply an empirical fitting parameter.

By incorporating the experimentally measured exponent into the Arrhenius formulation, the kinetic contribution associated with the temperature dependence of the degradation exponent is removed, producing a substantially more consistent lifetime extrapolation and a significantly smaller intrinsic activation energy. The resulting collapse of the experimental data demonstrates that much of the apparent variability originates from the degradation kinetics themselves rather than from measurement uncertainty alone.

A recently proposed thermodynamic interpretation based on Gibbs free energy and correlation entropy provides one possible physical explanation for these observations by relating the power–law exponent to the evolving degradation free energy. Whether this interpretation ultimately proves to be unique remains to be established. Nevertheless, the present experimental observations demonstrate that the measured exponent contains reproducible physical information that any successful microscopic model of BTI should ultimately explain.

Finally, the implications of this work extend beyond bias temperature instability. Many semiconductor reliability mechanisms exhibit empirical power–law degradation behavior, suggesting that the degradation exponent may provide an additional experimentally measurable quantity for evaluating competing physical models. The approach presented here therefore offers a practical method for extracting additional kinetic information from conventional reliability measurements while providing a more physically consistent basis for lifetime extrapolation.

## Data Availability

No new data were created or analyzed in this study. Data sharing is not applicable to this article.
